# Efficacy and safety of nitazoxanide plus atazanavir/ritonavir for the treatment of moderate to severe COVID-19 (NACOVID): A structured summary of a study protocol for a randomised controlled trial

**DOI:** 10.1186/s13063-020-04987-8

**Published:** 2021-01-04

**Authors:** Adeniyi Olagunju, Adeola Fowotade, Ajibola Olagunoye, Temitope Olumuyiwa Ojo, Bolanle Olufunlola Adefuye, Adeniyi Francis Fagbamigbe, Akindele Olupelumi Adebiyi, Omobolanle Ibitayo Olagunju, Olabode Taiwo Ladipo, Abdulafeez Akinloye, Babatunde Ayodeji Adeagbo, Adedeji Onayade, Oluseye Oladotun Bolaji, Christian Happi, Steve Rannard, Andrew Owen

**Affiliations:** 1 Department of Pharmaceutical Chemistry, Faculty of Pharmacy, Obafemi Awolowo University, Ile-Ife, Nigeria; 2 Department of Molecular and Clinical Pharmacology, University of Liverpool, Liverpool, United Kingdom; 3 Department of Medical Microbiology and Parasitology, College of Medicine, University of Ibadan, Ibadan, Nigeria; 4 State Specialist Hospital, Osogbo, Nigeria; 5 Department of Community Health, Faculty of Clinical Sciences, Obafemi Awolowo University Teaching Hospital, Ile-Ife, Nigeria; 6 Olabisi Onabanjo University Teaching Hospital, Sagamu, Nigeria; 7 Department of Epidemiology and Medical Statistics, Faculty of Public Health, University of Ibadan, Ibadan, Nigeria; 8 Department of Community Medicine, University of Ibadan, Ibadan, Nigeria; 9 Department of Surveillance and Epidemiology, Nigeria Centre for Disease Control, Abuja, Nigeria; 10 Oyo State Ministry of Health, Ibadan, Nigeria; 11 African Centre of Excellence for Genomics of Infectious Diseases, Redeemer’s University, Ede, Nigeria; 12 Department of Chemistry, School of Physical Sciences, University of Liverpool, Liverpool, United Kingdom

**Keywords:** COVID-19, SARS-CoV-2, nitazoxanide, atazanavir/ritonavir, randomised controlled trial, protocol

## Abstract

**Objectives:**

To investigate the efficacy and safety of repurposed antiprotozoal and antiretroviral drugs, nitazoxanide and atazanavir/ritonavir, in shortening the time to clinical improvement and achievement of SARS-CoV-2 polymerase chain reaction (PCR) negativity in patients diagnosed with moderate to severe COVID-19.

**Trial design:**

This is a pilot phase 2, multicentre 2-arm (1:1 ratio) open-label randomised controlled trial.

**Participants:**

Patients with confirmed COVID-19 diagnosis (defined as SARS-CoV-2 PCR positive nasopharyngeal swab) will be recruited from four participating isolation and treatment centres in Nigeria: two secondary care facilities (Infectious Diseases Hospital, Olodo, Ibadan, Oyo State and Specialist State Hospital, Asubiaro, Osogbo, Osun State) and two tertiary care facilities (Obafemi Awolowo University Teaching Hospitals Complex, Ile-Ife, Osun State and Olabisi Onabanjo University Teaching Hospital, Sagamu, Ogun State). These facilities have a combined capacity of 146-bed COVID-19 isolation and treatment ward.

**Inclusion criteria:**

Confirmation of SARS-CoV-2 infection by PCR test within two days before randomisation and initiation of treatment, age bracket of 18 and 75 years, symptomatic, able to understand study information and willingness to participate. Exclusion criteria include the inability to take orally administered medication or food, known hypersensitivity to any of the study drugs, pregnant or lactating, current or recent (within 24 hours of enrolment) treatment with agents with actual or likely antiviral activity against SARS-CoV-2, concurrent use of agents with known or suspected interaction with study drugs, and requiring mechanical ventilation at screening.

**Intervention and comparator:**

Participants in the intervention group will receive 1000 mg of nitazoxanide twice daily orally and 300/100 mg of atazanvir/ritonavir once daily orally in addition to standard of care while participants in the control group will receive only standard of care. Standard of care will be determined by the physician at the treatment centre in line with the current guidelines for clinical management of COVID-19 in Nigeria.

**Main outcome measures:**

Main outcome measures are: (1) Time to clinical improvement (defined as time from randomisation to either an improvement of two points on a 10-category ordinal scale (developed by the WHO Working Group on the Clinical Characterisation and Management of COVID-19 infection) or discharge from the hospital, whichever came first); (2) Proportion of participants with SARS-CoV-2 polymerase chain reaction (PCR) negative result at days 2, 4, 6, 7, 14 and 28; (3) Temporal patterns of SARS-CoV-2 viral load on days 2, 4, 6, 7, 14 and 28 quantified by RT-PCR from saliva of patients receiving standard of care alone versus standard of care plus study drugs.

**Randomisation:**

Allocation of participants to study arm is randomised within each site with a ratio 1:1 based on randomisation sequences generated centrally at Obafemi Awolowo University. The model was implemented in REDCap and includes stratification by age, gender, viral load at diagnosis and presence of relevant comorbidities.

**Blinding:**

None, this is an open-label trial.

**Number to be randomised (sample size):**

98 patients (49 per arm).

**Trial status:**

Regulatory approval was issued by the National Agency for Food and Drug Administration and Control on 06 October 2020 (protocol version number is 2.1 dated 06 August 2020). Recruitment started on 9 October 2020 and is anticipated to end before April 2021.

**Trial registration:**

The trial has been registered on ClinicalTrials.gov (July 7, 2020), with identifier number NCT04459286 and on Pan African Clinical Trials Registry (August 13, 2020), with identifier number PACTR202008855701534.

**Full protocol:**

The full protocol is attached as an additional file which will be made available on the trial website. In the interest of expediting dissemination of this material, the traditional formatting has been eliminated, and this letter serves as a summary of the key elements in the full protocol.

The study protocol has been reported in accordance with the Standard Protocol Items: Recommendations for Clinical Interventional Trials (SPIRIT) guidelines (Additional file 2).

**Supplementary Information:**

The online version contains supplementary material available at 10.1186/s13063-020-04987-8.

## Supplementary Information

**Additional file 1.** Full Study Protocol.

**Additional file 2.** SPIRIT 2013 Checklist: Recommended items to address in a clinical trial protocol and related documents.

## Data Availability

Trial data are available to the investigators through the Obafemi Awolowo University REDCap web platform. Summary data will be made open access. However, requests for access to disaggregated and anonymised data will be assessed on a case by case basis, depending on the qualifications of the requester, quality of request and type of secondary analysis proposed.

