# Supplementary material for: Efficacy and safety of nitazoxanide plus atazanavir/ritonavir for the treatment of moderate to severe COVID-19 (NACOVID): A structured summary of a study protocol for a randomised controlled trial
Source: Trials. 2021 Jan 4;22:3. doi: 10.1186/s13063-020-04987-8 (PMC7780204; doi:10.1186/s13063-020-04987-8)
Supplement: Supplementary file 1 — Additional file 1. Full Study Protocol. [file 13063_2020_4987_MOESM1_ESM.pdf]

NACOVID Trial

PROJECT TITLE: A randomised, open label trial to investigate the efficacy and safety of nitazoxanide plus atazanavir/ritonavir for the treatment of COVID-19: a pilot study

SHORT TITLE: The nitazoxanide plus atazanavir for COVID-19 study

CLINICAL TRIAL PHASE: Phase 2

CLINICALTRIALS.GOV IDENTIFIER: NCT04459286

PAN AFRICAN CLINICAL TRIALS REGISTRY IDENTIFIER: PACTR202008855701534

PROTOCOL NUMBER: 2.5

VERSION DATE: 02 October 2020

SPONSOR: Obafemi Awolowo University, Ile-Ife, Osun State, Nigeria

FUNDER: University of Liverpool, Liverpool, United Kingdom

ETHICS COMMITTEES: National Health Research Ethics Committee, Abuja, Nigeria and the University of Liverpool Health and Life Sciences Ethics Committee

ETHICS APPROVAL DATE: 26 August 2020 and 29 September 2020

ETHICS APPROVAL NUMBER: NHREC/01/01/2007-26/08/2020 and 8074

PRINCIPAL INVESTIGATOR: Adeniyi Olagunju, PhD
Senior Lecturer, Faculty of Pharmacy, Obafemi Awolowo University, Ile-Ife, Nigeria
Honorary Research Associate, Institute of Translational Medicine, University of Liverpool, Liverpool, United Kingdom
Mobile: +234906 885 8698
Email: aeolagunju@oauife.edu.ng or olagunju@liverpool.ac.uk

Confidentiality Statement

This clinical trial protocol is the property of Obafemi Awolowo University and the University of Liverpool. No part of this document, including all original concepts and ideas contained herein, may be reproduced, transmitted or disclosed in any form, or by any means, electronic or mechanical, for any purpose without the express written permission of Obafemi Awolowo University and the University of Liverpool, as represented by the Principal Investigator.

Table of Contents

List of Abbreviations 5
Investigators Signature Page 6
Statement of Compliance 7
Trial Synopsis 8
Schematic of Study Design 10
Key Roles 11
1. Detailed Description 13
1.1. Background 13
2. Rationale for Drug and Dose Selection 13
2.1. Nitazoxanide 14
2.2. Atazanavir/ritonavir 16
3. Study Design 16
3.1. Informed Consent Process, Recruitment and Randomisation 17
3.2. Trial Intervention 17
3.2.1. Dosing and Administration 17
3.3. Standard of care (SOC) 17
3.4. Follow-up 17
4. Safety Assessment 17
5. Outcome Measures 20
6. Power and Sample Size Determination 21
7. Study Sites and Population 21
7.1. Inclusion criteria 22
7.2. Exclusion criteria 22
8. Avoiding Risk of SARS-CoV-2 Transmission 22
9. Specimen Collection for SARS-CoV-2 Viral Load 23
10. Specimen Shipment and Storage 23
11. SARS-CoV-2 Detection and Viral Load Assay 23
12. Statistical Considerations 24
13. Assessments of Nitazoxanide Pharmacokinetics 24
14. Ethical Considerations 25
15. Protocol Amendment 25

16. Study Monitoring ... 26
16.1. Safety Review Committee (SRC) ... 26
16.2. Independent Medical Monitor ... 26
16.3. Independent Data and Safety Monitoring Committee (IDSMC) ... 26
17. Treatment Discontinuation and Withdrawal From Trial ... 26
17.1. Discontinuation ... 26
17.2. Withdrawal from the trial ... 27
18. Data Management ... 27
18.1. Electronic data capture ... 27
18.2. Source documentation ... 28
18.3. Data sharing ... 28
18.4. Archiving and retention of data ... 28
19. Adverse Events Management ... 28
19.1. Serious adverse event (SAE) ... 30
20. References ... 31
Appendix 1. Informed Consent Process ... 34
Appendix 2. Participant Information Leaflet ... 34
Appendix 3. Informed Consent Form – Method 1 ... 34
Appendix 3. Informed Consent Form – Method 2 ... 34
Appendix 4. Prescription Information ... 34
a) Nitazoxanide ... 34
b) Atazanavir/ritonavir ... 34
Appendix 5. Clinical Management of COVID-19 ... 34
Appendix 6. DAIDS Adverse Events Grading Table ... 34
Appendix 7. Liverpool Causality Assessment Tool (LCAT) ... 34
Appendix 8. The inFLUenza Patient-Reported Outcome (FLU-PRO) questionnaire ... 34
Appendix 9. Key Trial Contact Details ... 35

List of Abbreviations

ADR	Adverse Drug Reaction
AIDS	Acquired Immunodeficiency Syndrome
AKI	Acute Kidney Injury
ALT	Alanine Aminotransferase
ARDS	Acute Respiratory Distress Syndrome
AST	Aspartate Aminotransferase
BSL	Biosafety Level
COVID	Coronavirus Disease
CRF	Case Report Form
DAIDS	Division of Acquired Immunodeficiency Syndrome
DMSO	Dimethyl Sulfoxide
ECMO	Extracorporeal Membrane Oxygenation
FDA	US Food and Drug Administration
GCP	Good Clinical Practice
IATA	International Air Transport Association
ICH	International Conference for Harmonisation
IDSMC	Independent Data and Safety Monitoring Committee
LCAT	Liverpool Causality Assessment Tool
MHV	Mouse Hepatitis Virus
MOI	Multiplicity of Infection
NCDC	Nigeria Centre for Disease Control
NIV	Non-Invasive Ventilation
PCR	Polymerase Chain Reaction
PPE	Personal Protective Equipment
RNA	Ribonucleic Acid
SAE	Serious Adverse Event
SARS	Severe Acute Respiratory Syndrome
SOC	Standard of Care
SOP	Standard Operating Procedure
SRT	Safety Review Team
SUSAR	Suspected Unexpected Serious Adverse Reaction
ULN	Upper Limit of Normal
VTM	Viral Transport Medium
WHO	World Health Organisation

Investigators' Signature Page

A randomised, open label trial to investigate the efficacy and safety of nitazoxanide plus atazanavir/ritonavir for the treatment of COVID-19: a pilot study

I, the undersigned, am responsible for the conduct of the trial at the clinical site I represent and affirm that:

- I have read the protocol and agree that it contains all necessary details for carrying out the study as described.
- I agree to personally conduct the trial in compliance with GCP, with the applicable regulatory requirement(s), and with the protocol agreed to by the sponsor and given approval/favorable opinion by the ethics review committee.
- I have sufficient time to properly conduct and complete the trial within the agreed trial period, and I will ensure that any qualified staff at my site(s) who are involved in the trial conduct are adequately trained regarding the protocol and their responsibilities for the foreseen duration of the trial to conduct the trial properly and safely.
- Except where necessary to prevent immediate danger to the participant, I will not deviate from the protocol without prior written permission from the Sponsor and prior review and written approval from the Ethics Review Committee.
- I will comply with the procedure for data recording/reporting and retain essential documents for the duration specified in the approved protocol.
- I will permit monitoring, auditing, and inspection of trial related activities and documentation by the sponsor.
- I agree to ensure that all associates, colleagues and employees assisting in the conduct of the study are informed about their obligations in meeting the above commitments.
- I understand that the study may be terminated, or enrolment suspended at any time by the Sponsor, with or without cause, or by me if it becomes necessary to protect the best interest of the participants.

**Investigators**	**Signature**
Adeniyi Olagunju, PhD	
Dr Adeola Fowotade, MBBS, MSc, PhD	
Dr Temitope Ojo, MBChB, MPH	
Dr Ajibola Olagunoye, MBBS, MPH, FEP, FWACP	
Dr Bolanle Adefuye, MBBS, FWACP, MSc	
Dr Adeniyi Francis Fagbamigbe, PhD	
Dr Akindele Olupelumi Adebiyi, MBChB, PhD, FMCPH	
Omobolanle Olagunju, MSc	
Dr Olabode Ladipo, MBBS	
Dr Babatunde Adeagbo, PhD	
Prof. Adedeji Onayade, MBBS, MPH, FWACP, FMCPH	
Professor Christian Happi, PhD	
Professor Oluseye Bolaji, PhD	
Professor Steve Rannard, PhD	
Professor Andrew Owen, PhD	

Statement of Compliance

The trial will be conducted in accordance with the International Conference on Harmonisation (ICH) E6 Good Clinical Practice (GCP), Declaration of Helsinki, the Nigeria National Code of Health Research Ethics and the Terms of Award. The Principal Investigator will assure that no deviation from, or changes to the protocol will take place without prior agreement from the sponsor and documented approval from the Research Ethics Committee, except where necessary to eliminate an immediate hazard(s) to the trial participants. All personnel involved in the conduct of this study have completed Human Subjects Protection Training.

I agree to ensure that all staff members involved in the conduct of this study are informed about their obligations in meeting the above commitments.

**Principal Investigator: (Print Full Name)**	Adeniyi Olagunju
**Signature:**	
**Date:**	

Trial Synopsis

**Official Title**	A randomised, open label trial to investigate the efficacy and safety of nitazoxanide plus atazanavir/ritonavir for the treatment of COVID-19: a pilot study
**Short Title**	The nitazoxanide plus atazanavir for COVID-19 study
**Trial Registration Number**	NCT04459286
**PACTR Registration Number**	PACTR202008855701534
**Background**	COVID-19 caused by SARS-CoV-2 is an unprecedented global public health challenge which as at 24 May 2020 has spread to over 210 countries with over 5.31 million cases including 342,000 deaths. More than 1130 clinical trials are currently ongoing in an unprecedented global search for potential therapeutics and vaccines.
**Objectives**	The purpose of this phase 2 trial is to investigate the efficacy and safety of repurposed antiprotozoal and antiretroviral drugs, nitazoxanide and atazanavir/ritonavir, in achievement of SARS-CoV-2 PCR negativity and shorten the time to clinical improvement in patients diagnosed with moderate to severe COVID-19.
**Recruitment**	Individuals between 18 and 75 years old who receive a PCR positive test for COVID-19 and admitted at participating COVID-19 treatment centres will be invited to participate.
**Sample Size**	98
**Randomisation**	For this pilot study, consenting individuals will be randomised 1:1 to receive either standard of care (SOC) alone (n = 49) or SOC plus daily trial intervention (n = 49).
**Intervention**	Arm 1: SOC<br>Arm 2: SOC + 1000 mg nitazoxanide twice daily and 300/100 mg atazanavir/ritonavir once daily with meal for 14 days

Objectives	Outcome Measures

**Primary Objectives and Outcome Measures**	• To investigate time to clinical improvement in patients receiving study drug plus SOC compared with patients receiving SOC alone.	Time to clinical improvement (defined as time from randomization to either an improvement of two points on a 10-category ordinal scale or discharge from the hospital, whichever came first).
	• To compare time to SARS-CoV-2 negativity between study drug plus SOC and SOC alone.	Proportion of participants with SARS-CoV-2 polymerase chain reaction (PCR) negative result at Days 7, 10, 14 and 28.
	• To test the efficacy of study drug plus SOC compared to SOC alone in reducing SARS-CoV-2 viral load.	Temporal patterns of SARS-CoV-2 viral load quantified by RT-PCR from nasal swabs or sputum of patients receiving SOC alone versus SOC plus study drug..
**Ethics**	This trial will be conducted in compliance with ICH Guideline E6 for Good Clinical Practice and all applicable regulations. Ethical clearance will be obtained from the National Health Research Ethics Committee, Nigeria.	
**Sites Enrolling Participants**	1. Infectious Diseases Hospital, Olodo, Ibadan, Oyo State<br>2. Obafemi Awolowo University Teaching Hospital, Ile-Ife, Osun State<br>3. State Specialist Hospital, Asubiaro, Osogbo, Osun State<br>4. Olabisi Onabanjo University Teaching Hospital, Sagamu, Ogun State	
**Description of Study Intervention**	Combination therapy composed of **nitazoxanide** (an antiprotozoal drug approved for the treatment of diarrhea caused by *Giardia lamblia* or *Cryptosporidium parvum*) and **atazanavir/ritonavir** (HIV protease inhibitor approved for the treatment of HIV) shown to have in vitro activity against SARS-CoV-2.	
**Study Duration**	3-6 Months	
**Participant Duration**	1 Month	

Schematic of Study Design

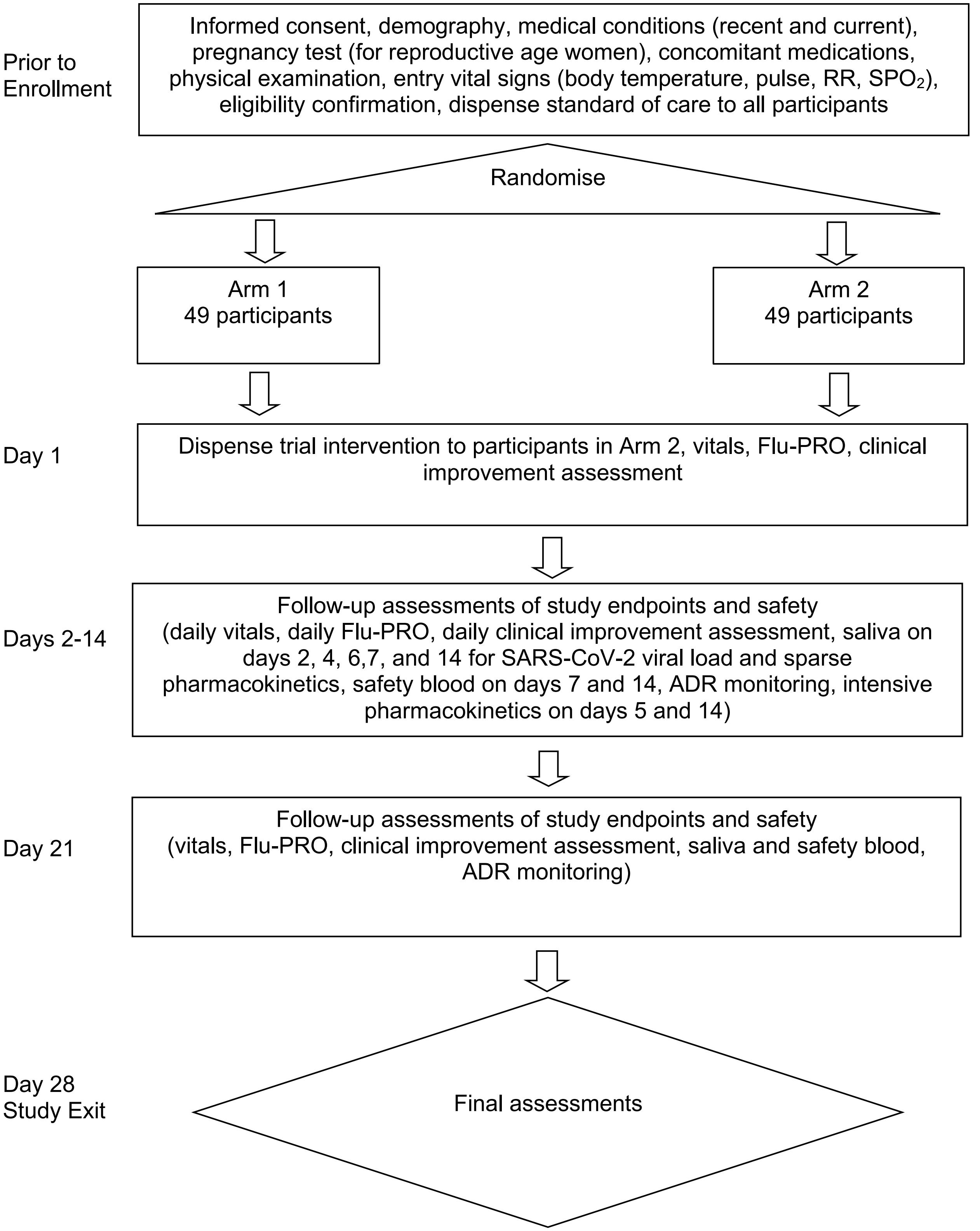

Key Roles

Adeniyi Olagunju, PhD<br>Senior Lecturer	Faculty of Pharmacy, Obafemi Awolowo University, Ile-Ife; and Institute of Translational Medicine, University of Liverpool, Liverpool, United Kingdom	Principal Investigator
Adeola Fowotade, MBBS, MSc and PhD<br>Clinical Virologist	Infectious Disease Hospital, Olodo, Ibadan, Oyo State, Nigeria	Clinical Investigator
Temitope Ojo, MBChB<br>Consultant Community Physician	Obafemi Awolowo University Teaching Hospital, Ile-Ife, Osun State, Nigeria	Clinical Investigator
Ajibola Olagunoye, MBChB<br>Consultant and Public Health Physician; State COVID-19 Case Manager	State Specialist Hospital, Asubiaro, Osogbo, Osun State, Nigeria	Clinical Investigator
Bolanle Adefuye, MBBS FWACP, MSc<br>Consultant Pulmonologist	Olabisi Onabanjo University Teaching Hospital, Sagamu, Ogun State, Nigeria	Clinical Investigator
Adeniyi Francis Fagbamigbe, PhD<br>Senior Lecturer	Department of Epidemiology and Medical Statistics, Faculty of Public Health, College of Medicine, University of Ibadan, Ibadan, Oyo State	Biostatistician
Akindele Olupelumi Adebiyi, MBChB, PhD, FMCPH<br>Consultant Community Physician and Clinical Epidemiologist	Department of Community Medicine, Faculty of Clinical Sciences, College of Medicine, University of Ibadan, Oyo State, Nigeria	Clinical Epidemiologist
Omobolanle Olagunju, MSc<br>Senior Scientific Officer	Department of Surveillance and Epidemiology, Nigeria Centre for Disease Control, Ebitu Ukiwe Street, Jabi, Abuja	Infection Prevention and Control Scientist
Olabode Ladipo, MBBS<br>Satte COVID-19 Incident Manager	Oyo State Ministry of Health, Ibadan, Oyo State, Nigeria	Public Health Physician
Babatunde Adeagbo, PhD<br>Senior Lecturer	Translational Pharmacokinetics Research Laboratory, Faculty of Pharmacy, Obafemi Awolowo University, Ile-Ife, Nigeria	Pharmacokineticist
Adedeji Onayade, MBBS, MPH, FWACP, FMCPH<br>Professor and Consultant Community Physician	Obafemi Awolowo University Teaching Hospital, Ile-Ife, Osun State, Nigeria	Community Physician
Christian Happi, PhD<br>Professor	African Centre of Excellence for Genomics of Infectious Diseases, Redeemer's University, Ede, Nigeria	Molecular Biologist

Oluseye Bolaji, PhD Professor	Translational Pharmacokinetics Research Laboratory, Faculty of Pharmacy, Obafemi Awolowo University, Ile-Ife, Nigeria	Pharmacokineticist
Steve Rannard, PhD Professor	Department of Chemistry, School of Physical Sciences, University of Liverpool, UK	Drug Repurposing Chemist
Andrew Owen, PhD Professor	Department of Molecular and Clinical Pharmacology, Institute of Translational Medicine, University of Liverpool, UK	Pharmacologist

1. Detailed Description

1.1. Background

Health authorities in the Chinese city of Wuhan identified a cluster of pneumonia cases of unknown aetiology in December 2019 initially linked to the city's seafood market known as the Huanan Seafood Wholesale Market[1]. Subsequent investigations revealed the causative agent as a novel coronavirus, severe acute respiratory syndrome coronavirus (SARS-CoV-2)[2]. SARS-CoV-2 is now at the heart of what is now a global coronavirus disease (COVID-19) pandemic[3]. An unprecedented global public health action was launched to stop the spread. But as at 24 May 2020 it has spread to over 210 countries with over 5.31 million cases including 342,000 deaths. More than 1130 clinical trials are currently ongoing in an unprecedented global search for potential therapeutics and vaccines.

Though there is currently no proven effective therapies, increasing understanding of SARS-CoV-2 virology provides a significant number of potential drug targets[4]. Early results from some of the trials have been mixed, with some studies reporting no benefits while some found potential benefits. For instance, among 1438 patients hospitalized in metropolitan New York with COVID-19, there was no difference in in-hospital mortality for treatment with hydroxychloroquine alone (19.9%, 54/271), azithromycin alone (10.0%, 21/211), or both (25.7%, 189/735), compared with neither treatment (12.7%, 28/221)[5]. A separate retrospective analysis reported significantly lower fatalities in critically ill patients who received hydroxychloroquine on top of basic treatments with antivirals and antibiotics compared with those on basic treatments alone (18.8% (9/48) versus 47.4% (238/502)), possibly through attenuation of inflammatory cytokine storm[6]. Reports from three large trials (as well a now retracted paper[7]) have now confirmed that hydroxychloroquine is not effective for COVID-19 postexposure prophylaxis[8] or treatment[9]. Importantly, more than 20% of ongoing trials (230/1093) are evaluating chloroquine or hydroxychloroquine.

Preliminary results from remdesivir compassionate use programme in a cohort of patients hospitalized for severe COVID-19 indicate clinical improvement in 68% of patients (36/53)[10]. Triple antiviral therapy with interferon beta-1b, lopinavir/ritonavir and ribavirin resulted in SARS-CoV-2 clearance, symptoms resolution and shorter hospital stay than lopinavir/ritonavir alone (7 versus 12 days, hazard ratio 4·37, p=0·0010) in patients with mild to moderate COVID-19 in an open-label, randomised, phase 2 trial[11]. While these results seem promising, definitive data to guide the management of COVID-19 patients are still pending. Additionally, daily intravenous infusion is not a global solution compatible with the healthcare systems in all country contexts. Results from more than 90 vaccine candidates are expected later in 2020 and early 2021.

With increasing number of cases reported in low- and middle-income countries and south America[12], and the possibility of a second wave of infections in countries where the pandemic appears to have slowed, studies to investigate other promising therapies are urgently needed, especially those that can significantly reduce time to viral clearance and mortality. Shortening SARS-CoV-2 clearance time will lower treatment cost and reduce the economic impact of an extended isolation period.

2. Rationale for Drug and Dose Selection

The selection of candidates for this COVID-19 drug repurposing trial was guided by three pharmacological considerations[13]: (1) demonstration of in vitro anti-SARS-CoV-2 activity at doses shown or predicted to be tolerated by humans, (2) the feasibility of achieving effective concentration in relevant compartments, and (3) established human safety record.

2.1. Nitazoxanide

1. Demonstration of in vitro anti-SARS-CoV-2 activity: A review of *in vitro* studies reporting the anticoronavirus activity of nitazoxanide and its active metabolite, tizoxanide, is available[14]. Out of 727 compounds in the NIH Clinical Collection small molecule library, 84 drugs had significant anti-coronavirus activity, including 51 entry blockers and 19 inhibitors of viral replication. Nitazoxanide was among the three top inhibitors, resulting in a reduction of 6 log_{10} in virus titer with an IC_{50} of 1.0 µM[15]. The major circulating metabolite of nitazoxanide is tizoxanide and recent work by the NIH National Centre for Advancing Translational Sciences have also confirmed its activity against SARS-CoV-2[16]. Both suppress the cytopathic effect of SARS-CoV-2. Nitazoxanide is also active against the influenza A virus and was shown to reduce symptoms duration in acute uncomplicated influenza[17]. The susceptibility of 210 seasonal influenza viruses to nitazoxanide and its metabolite tizoxanide has been reported[18]. Since SARS-CoV-2 shares almost 80% of the genome with SARS-CoV[19] and almost all encoded proteins of SARS-CoV-2 are homologous to SARS-CoV proteins[20], nitazoxanide and its metabolite tizoxanide with demonstrated activity against SARS-CoV are likely to be effective against SARS-CoV-2.

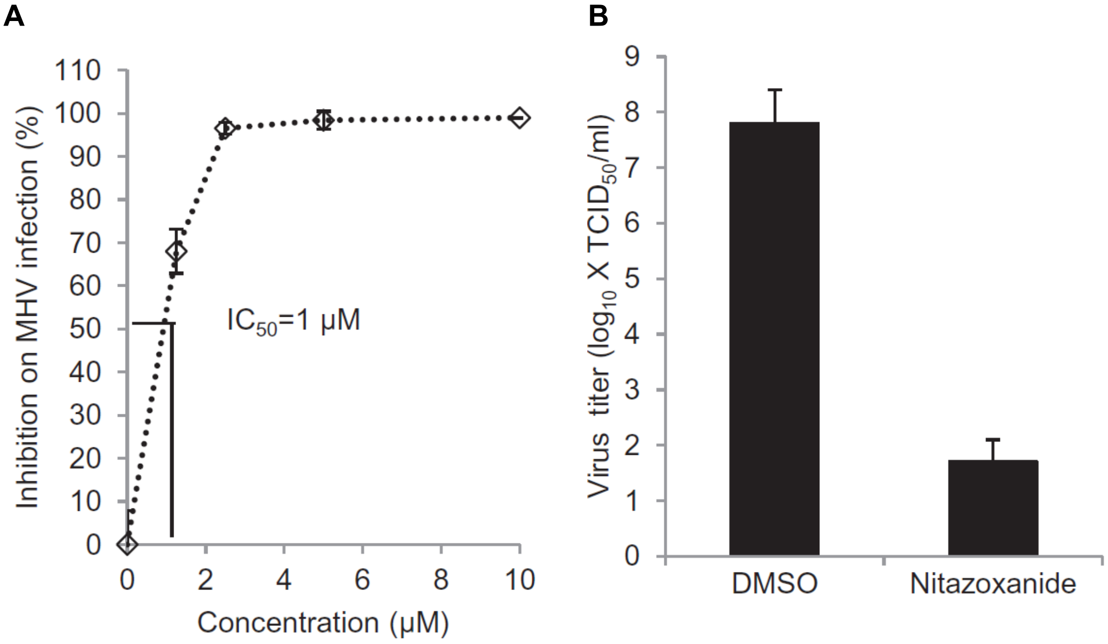

Figure 1. Inhibitory effect of nitazoxanide on murine coronavirus mouse hepatitis virus (MHV) infection. (A) Determination of IC_{50}. Mouse astrocytoma DBT cells were treated with nitazoxanide at various concentrations or 1% dimethyl sulfoxide (DMSO; vehicle control) for 1 h, and were infected with MHV strain A59 expressing firefly luciferase (MHV-2aFLS) at multiplicity of infection (MOI) of 1 in the presence of the drug for 8 h. Cells were then lysed for luciferase assay. Inhibition

of drug on MHV infection was expressed as percent reduction on luciferase activity to the control and the IC_{50} was then calculated as indicated by the solid lines. (B) Inhibition of viral titer. DBT cells were treated with nitazoxanide (10 μM) or DMSO (1%) as a control for 1 h and then infected with MHV-2aFLS at MOI of 1 for 12 h. The medium was harvested for determining viral titer (Tissue Culture Infectious Dose, $TCID_{50}$). Data indicate the mean of 3 replicates and standard deviation of the mean. Source: Cao et al 2015[15].

2. Feasibility of achieving effective concentration in relevant compartments: In a comprehensive analysis involving 56 approved drugs with reported anti-SARS-CoV-2 activity, nitazoxanide was among the only 12 predicted to achieve effective concentration in the lungs throughout the dosing interval[21]. However, the usually dose of 500 mg twice daily for diarrhea was shown to be inadequate in achieving minimum effective concentration in the lungs and the systemic circulation (Figure 2). Since viruses replicate quickly, it is important that antivirals remain active across their dosing interval, and a follow-up model-based dose optimization study was used to predict the minimum lung and plasma concentrations of tizoxanide for different dosing regimens of nitazoxanide.[22]

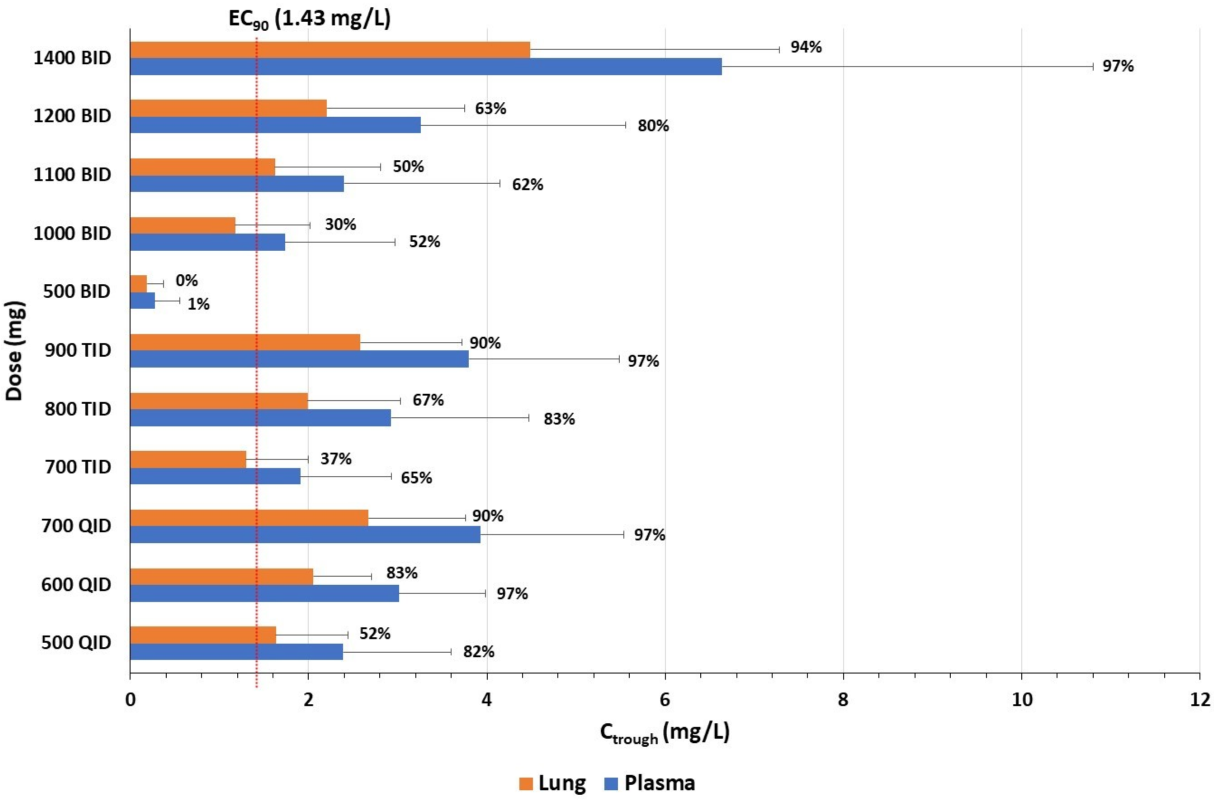

Figure 2 Rational for nitazoxanide dose selection. C_{trough} is predicted tizoxanide minimum concentration for various dosing regimens of nitazoxanide in fed state at the end of the first dose (BID – 12 h, TID – 8 h, QID – 6 h). Data is presented as mean and error bars represent standard deviation. The percentages adjacent to the bar chart indicate the percentage of simulated population over EC_{90} of nitazoxanide for SARS-CoV-2. Source: Rajoli et al 2020[22].

Therefore, a dosing regimen of 1000 mg nitazoxanide with meal two times daily for 14 days is expected to provide a balance of higher pulmonary drug exposure within doses that have been shown to be safe in humans. Thus, this dose has been chosen for this trial.

3. Established safety record: Nitazoxanide is an antiprotozoal drug with a well understood and documented safety profile. It was first approved by the US Food and Drug Administration (FDA) in July 2004 for the treatment of diarrhea caused by *Giardia lamblia* or *Cryptosporidium parvum* in adults (≥ 18 years) and paediatrics (1-17 years). Safety information from 1628 adults and adolescents who received nitazoxanide tablets in controlled and uncontrolled studies reported no death and adverse events included abdominal pain (6.7%), diarreah (4.3%), headache (3.1%), nausea (3.1%) and dizziness (1%)[23]. No grade 4 or 5 adverse events were reported. Single oral doses of up to 4000 mg nitazoxanide have been administered to healthy adult volunteers without significant adverse effects[24]. A 1000 mg twice daily dosage for 14 days has been reported and found to be safe[25,26]. No safety issues were identified at doses up to 3000 mg/day for a median of 62 days (range: 1-1528) in AIDS-related cryptosporidiosis[27]. Though no evidence of teratogenicity was observed in animals treated with multiple times the clinical adult dose adjusted for body surface area, nitazoxanide safety during human pregnancy or lactation has not been evaluated. Also, it has not been studied in patients with renal or hepatic impairment.[23]

2.2. Atazanavir/ritonavir

The HIV protease inhibitor, atazanavir (boosted with ritonavir), has been shown to inhibit the major protease enzyme required for viral polyprotein processing during coronavirus replication[28,29]. It also blocks pro-inflammatory cytokine production[28]. Similar to nitazoxanide, atazanavir has been shown to achieve effective concentration pulmonary tissues at approved dose of 300 mg in combination with 100 mg of ritonavir[21], with even more favourable ratios when compared to *in vitro* activities generated in human cell models[28]. Hence, it is a good candidate for drug repurposing in COVID-19. Additionally, tizoxanide is inactivated by glucuronidation and atazanavir is a well-known inhibitor[30]. Hence, when used in combination with nitazoxanide, atazanavir is expected to enhance tizoxanide exposure.

Therefore, based on these considerations, the combination of nitazoxanide (taken orally at 1000 mg twice daily) and atazanavir/ritonavir (taken orally at 300/100 mg once daily) is selected as the intervention for this trial.

3. Study Design

This is a pilot phase 2, open label randomised controlled trial. A total of 98 patients with confirmed COVID-19 diagnosis (defined as SARS-CoV-2 polymerase chain reaction (PCR) positive nasopharyngeal swab) will be recruited from participating treatment centres. Participants will be randomised to receive either the standard of care (SOC) plus nitazoxanide for 14 days, starting from day 1 or the SOC alone for 14 days.

3.1. Informed Consent Process, Recruitment and Randomisation

Participants will be recruited within 2 days of admission into COVID-19 treatment centre. The informed consent process is presented in Appendix 1. It is based on the recently published FDA Guidance on Conduct of Clinical Trials of Medical Products during COVID-19 Public Health Emergency[31] and will ensure compliance with both GCP regulations and infection prevention and control policy at trial sites. Before enrollment, they will be given adequate information about the trial (Appendix 2), opportunity to ask questions, and sufficient time to consider participation. Before any screening procedures, an informed consent will be obtained from each eligible subject who agrees to participate in the trial (Appendix 3). This will be conducted by the clinical co-investigator or delegated healthcare personnel on site and implemented electronically using Research Electronic Data Capture (REDCap). Allocation of participants to study arm will be randomised within each site with a ratio 1:1 based on randomisation sequences generated centrally at Obafemi Awolowo University. The model will be implemented in REDCap and will include stratification by age, gender, viral load at diagnosis and presence of relevant comorbidities.

3.2. Trial Intervention

The full prescription information for nitazoxanide tablet is presented in Appendix 4(a) and that of atazanavir/ritonavir is presented in Appendix 4(b).

3.2.1. Dosing and Administration

Participants in the intervention group will receive 1000 mg nitazoxanide (two tablets of 500 mg each) with meal two times daily (8 am and 8 pm) and one tablet of 300/100 mg atazanavir/ritonavir with meal once daily (8 pm) in addition SOC.

3.3. Standard of care (SOC)

SOC will be as determined by the clinical team at the treatment centres in line with the current National Interim Guidelines for Clinical Management of COVID-19[32] (Appendix 5).

3.4. Follow-up

The treatment duration for participants in the intervention group will be 14 days. However, follow up will continue until day 28 after study entry at the end of which all participants will exit the study. The schedule of activities during the follow up period is presented in Table 1. Participants who stop study product should continue study participation off study product with continued evaluations as per the schedule of activities. The reason for study product discontinuation should be recorded. Daily survey, vital signs and SpO2 as well as Flu-PRO questionnaire will be recorded by participant (both on paper and cloud-based forms using their mobile device while connected to the internet). SpO2 will be measured after at least 5 minutes resting in a sitting or supine position. Participants with an SpO2 below 93% will repeat the assessment 2 hours later. If the repeat assessment is still below 93%, study clinician will be alerted.

4. Safety Assessment

Participants will be assessed for adverse events daily throughout study period. Each morning the attending physician will ask about the side effects commonly associated of nitazoxanide (including gastrointestinal symptoms and headaches) and any additional symptoms using a structured questionnaire. Staff at the treatment centre will further assess participants for the occurrence of new symptoms at least every six hours. Adverse events will be graded according to the NIH Division of AIDS grading scale (Appendix 6) and causality will be determined using Liverpool Causality Assessment Tool (Appendix 7). All serious adverse events, including those resulting in discontinuation of trial intervention, whether or not they are judged to be related to the trial, will be recorded as per trial schedule of follow up activities. All safety issues will be reviewed after the first 15 participants in the intervention group have completed 14 days of follow up and when any grade 3 or higher adverse event occurred in the intervention group.

Table 1. Schedule of Follow Up Activities

**Procedure**	**Day 0**	**Day 1**	**Day 2**	**Day 3**	**Days 4-6**	**Day 7**	**Days 8-9**	**Day 10**	**Days 11-13**	**Day 14**	**Day 21**	**Day 28**
Informed consent	X											
Demography	X											
Medical history (recent and current)	X											
Pregnancy test (for reproductive age women)	X											
Concomitant medications	X											
Physical examination	X											
Entry vital signs	X											
Eligibility confirmation	X											
Enrolment	X											
Safety blood (10 mL)[1]	X					X				X		
Dispense standard of care	X											
Randomisation	X											
Dispense trial intervention (BID for 14 days)		X	X	X	X	X	X	X	X	X		
Daily vitals, including SpO_2		X	X	X	X	X	X	X	X	X	X	X
Flu-PRO		X	X	X	X	X	X	X	X	X	X	X
Clinical improvement assessment		X	X	X	XXX	X	XX	X	XXX	X	X	X
Saliva or swab collection for SARS-CoV-2[2]	X		X		X	X				X		X
Pharmacokinetic sampling[3]			X		X**X**X	X				X**X**		
Adverse event monitoring		X	X	X	X	X	X	X	X	X	X	X
Study exit assessment												X

[1] 10 mL blood draws each at baseline, day 7 and day 14 will be used to assess essential biochemical (including AST, ALT) and haematology (including XXX) safety parameters.

[2] Sampling schedule for SARS-CoV-2 viral load: Days 0, 2, 4, 6, 7, 14, and 28.

[3] Sparse PK sampling in all patients paired with saliva sample for SARS-CoV-2 viral load, as well as intensive PK sampling on days 5 and 14 in 12 selected participants.

5. Outcome Measures

Primary outcome measures:

- Time to clinical improvement (defined as time from randomization to either an improvement of two points on a 10-category ordinal scale[33] or discharge from the hospital, whichever came first):[4]

Patient State	Descriptor	Score
Uninfected	Uninfected; no viral RNA detected	0
Ambulatory (Mild Disease)	Asymptomatic; viral RNA detected	1
	Symptomatic; independent	2
	Symptomatic; assistance needed	3
Hospitalised (Moderate Disease)	Hospitalized, not oxygen therapy	4
	Hospitalized; oxygen by mask or nasal prongs	5
Hospitalised (Severe Disease)	Hospitalized, oxygen by NIV or high flow	6
	Intubation and mechanical ventilation, $PaO_2/FiO_2 \geq 150$ or $SpO_2/FiO_2 \geq 200$	7
	Mechanical ventilation $PaO_2/FiO_2 < 150$ ($SpO_2/FiO_2 < 200$) or vasopressors	8
	Mechanical ventilation $PaO_2/FiO_2 < 150$ and vasopressors, dialysis, or ECMO	9
Dead	Dead	10

Abbreviations: ECMO, Extracorporeal membrane oxygenation; NIV, Non-invasive ventilation; FiO2, Fraction of inspired oxygen; PaO_2, pressure of arterial oxygen; SpO_2, oxygen saturation

- Time to PCR negativity. Proportion of participants with SARS-CoV-2 polymerase chain reaction (PCR) negative result at Days 7, 10, 14 and 28.
- Temporal patterns of SARS-CoV-2 viral load quantified by RT-PCR from nasal swabs or sputum of patients receiving SOC alone versus SOC plus study drug.

Secondary outcome measures:

- Time to symptoms resolution as monitored by the inFLUenza Patient-Reported Outcome (FLU-PRO) questionnaire[34] with some modifications for COVID-19[35] (Appendix 8).
- Clinical status as assessed with the 10-category ordinal scale on days 7 and 14
- Duration of hospitalization in survivors
- Day 28 mortality
- Time from treatment initiation to death
- Proportion with viral RNA detection over time

[4] Note: PaO_2/FiO_2 of 200-300, mild ARD; 100-200, moderate ARD; <100, severe ARD

Safety outcome measures:

- Adverse events during treatment
- Serious adverse events (respiratory failure or Acute Respiratory Distress Syndrome (ARDS), acute kidney Injury (AKI), secondary infection, shock, severe anemia, acute gastritis, unconsciousness, sepsis, acute heart failure)
- Gastrointestinal adverse events (nausea, vomiting, and diarrhea)
- Premature discontinuation of treatment

6. Power and Sample Size Determination

As shown in Figure 3, the sample size estimation is based on the assumption that an improvement of at least 60-80% in time to SARS-CoV-2 PCR negativity and symptoms resolution can be achieved in the intervention group compared with the control group. Hence, a total sample size of 89 will provide at least 80% power to show or exclude 60% improvement in time to SARS-CoV-2 PCR negativity. This assumes a two-sided and 5% type 1 error rate. Therefore, providing for a 10% loss to follow up rate, a total of 98 patients will be recruited.

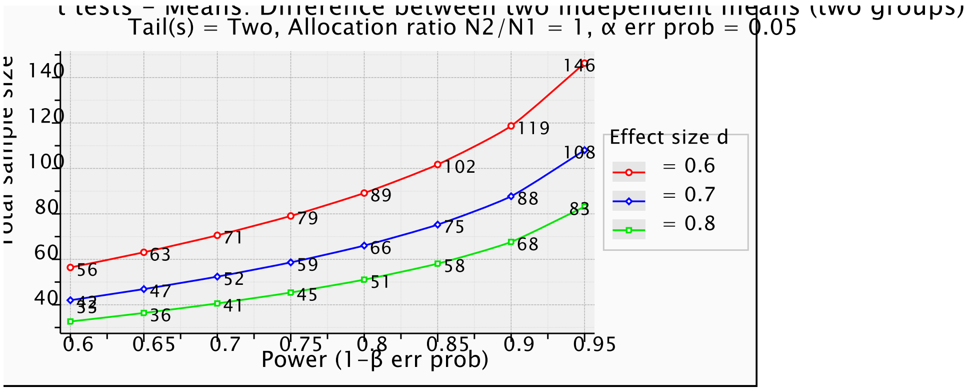

Figure 3. Power calculation and sample size estimation.

7. Study Sites and Population

Individuals (female or male) with confirmed COVID-19 test confirmed by PCR will be identified from the following isolation and treatment centres in Nigeria:

- Infectious Diseases Hospital, Olodo, Ibadan, Oyo State

- Obafemi Awolowo University Teaching Hospital, Ile-Ife, Osun State
- Specialist State Hospital, Asubiaro, Osogbo, Osun State
- Olabisi Onabanjo University Teaching Hospital, Sagamu, Ogun State

Eligibility will be ascertained using the following inclusion and exclusion criteria:

7.1. Inclusion criteria

- Willingness and ability to provide written informed consent.
- At least 18 and not more than 75 years of age at study entry.
- SARS-CoV-2 infection confirmed by PCR test within 2 days before randomization.
- Currently symptomatic (fever or chills, cough, myalgia, sore throat, shortness of breath, or new onset of anosmia or ageusia) and at COVID-19 isolation and treatment centre.

7.2. Exclusion criteria

- Inability to take orally administered medication or food.
- Known hypersensitivity to study medication.
- Pregnant or lactating (unless practicing exclusive replacement feeding for the entire study duration).
- Participation in any other interventional trial for COVID-19 (observational study co-enrollment allowed).
- Concurrent treatment with other agents with actual or possible direct-acting antiviral activity against SARS-CoV-2 less than 24 hours prior to study drug dosing.
- Concurrent use of agents with known or uncertain interaction with study drugs, including ritonavir.
- Requiring mechanical ventilation at screening.

8. Avoiding Risk of SARS-CoV-2 Transmission

To avoid the risk of SARS-CoV-2 transmission in connection with this trial, the following strategies will be adopted:

- Participants will be recruited only from government approved isolation and treatment centres which have been specially provisioned to safely care for COVID-19 patients and restrict contact with uninfected individuals.
- All procedures involving specimen collection, processing and analysis will be carried out in appropriately equipped facilities by staff trained in the relevant technical and safety procedures and in line with national/international guidelines on personal protective equipment and laboratory biosafety.
- Study personnel will wear personal protection equipment (PPE) at all times during any necessary physical interactions. Both participants and study personnel will be instructed to adhere to national and regional guidance for COVID-19-related safety measures.
- To further limit exposure, daily vital signs and symptoms will be self-monitored by participants whenever possible.

9. Specimen Collection for SARS-CoV-2 Viral Load

Saliva will be self-collected by participants under supervision into 15 mL screw-cap tubes from which 1.5-2mL will be transferred into 2 mL cryo vials for SARS-CoV-2 viral load determination. Where necessary nasopharyngeal swab specimen will be collected using Dacron® or Copan® polyester flocked swabs. Insert flexible wire shaft minitip swab through the nares parallel to the palate (not upwards) until resistance is encountered or the distance is equivalent to that from the ear to the nostril of the patient, indicating contact with the nasopharynx. Swab will reach a depth equal to distance from nostrils to outer opening of the ear and gently rubbed and rolled. Swab will be left in place for at least 15 seconds to absorb secretions and then slowly removed while being rotated.

10. Specimen Shipment and Storage

Samples will be stored at 4°C for analysis within 24 hours after collection. If a delay in testing or shipping is expected, specimens will be stored at -70°C or lower. Each swab will be placed in sterile tube containing 2-3 mL of VTM containing antifungal and antibiotic supplements. Repeated freezing and thawing of specimens will be avoided. Sterile saline will be used in the absence of VTM if samples will be stored at -70°C and analysed within 72 hours. Specimens will be packaged, shipped, and transported according to the current edition of the International Air Transport Association (IATA) Dangerous Goods Regulations [3].

11. SARS-CoV-2 Detection and Viral Load Assay

Handling of COVID-19 swab specimen for molecular testing will only be carried out in Nigerian Centre for Disease Control (NCDC) accredited BSL-2 or equivalent facilities. SARS-CoV-2 detection and viral load assays will be conducted at the African Centre of Excellence for Genomics of Infectious Diseases (ACEGID), Redeemer's University, Ede, Osun State Nigeria. SARS-CoV-2 RNA will be extracted using QIAamp Viral RNA Mini Kit based on the manufacturer protocol (QIAGEN, 2020). Approximately 60μL of RNA template will be extracted from 140μL of the specimen. One-step quantitative reverse transcriptase polymerase chain reaction (q RT-PCR) technique with fluorescent probes will be used for the molecular diagnosis and quantification of SAR-CoV 2. The protocol to be used will be based on the methodology of DAAN Gene extraction kit (Anon, 2020). Briefly, 5μL of the RNA template, negative control (NC) and positive control (PC) will be analysed in a total reaction volume of 25μL. The reaction mixture in the 96 well plate tubes will be tightly covered, vortex gently, centrifuged at 8,000 rpm for several seconds and amplified based on the manufacturer reaction condition as stated below.

Step	Cycles	Target Temperature	Running time
1	1	50°C	15 min
2	1	95°C	15 min
3	45	94°C	15 sec
		55°C	45 sec

The cycle threshold values of rRT-PCR will be used as indicators of the copy number of SARS-CoV-2 RNA in specimens with lower cycle threshold values corresponding to higher viral copy numbers. A cycle threshold value less than 40 is interpreted as positive for SARS-CoV-2 RNA. SARS-CoV-2 viral load in sputum and/or swab specimens will be determined as previously described.

12. Statistical Considerations

Proportion of participants with SARS-CoV-2 PCR negative result at days 7, 14 and 28 will be compared between the intervention and control groups. Analysis of clinical improvement based on the 10-category ordinal scale[33] will be performed using time-to-event data (patients will be censored at 28-days of follow-up). Categorical variables will be analysed using the log-rank test and continuous variables will be assessed using a univariable Cox proportional hazard regression analysis. Analysis of cumulative improvement rate will be presented by Kaplan-Meier survival curves when independent variables are dichotomous or categorical. A univariable Cox regression analysis will be conducted and covariates with a of $p < 0.25$ in the univariable analysis will be included in the multivariable model. These analyses will be conducted using Stata®.

An interim analysis of data from the Pilot Stage will be conducted after the first 30 participants have completed days 1-14 of the study period. These data will provide valuable insights regarding the design, conduct and analysis of the Pivotal Stage, including sample size estimate, intervention-responsive definition of the primary and secondary endpoints, and procedures to enhance quality of trial conduct. The Trial Steering Committee will make timely recommendations for finalizing the design of the Pivotal Stage based on data from the Pilot Stage. To preserve the integrity of the Pivotal Stage, data from the Pilot Stage would only be included in the primary analyses of the Pivotal Stage data only if those using the Pilot Stage data to enlighten decisions about finalizing the design of the Pivotal Stage do not have access to information from the Pilot Stage that would be directly or indirectly informative about the efficacy and safety of the experimental regimens being evaluated in the Pivotal Stage [4].

13. Assessments of Nitazoxanide Pharmacokinetics

Following oral administration of 500 mg of nitazoxanide every 12 hours for 7 consecutive days in adults, the mean (SD) C_{max} of tizoxanide was 10.6 (2.1) µg/mL and no significant accumulation of nitazoxanide metabolites tizoxanide or tizoxanide glucuronide was detected[23]. In a study that investigated nitazoxanide efficacy for pulmonary tuberculosis, a 1000 mg twice daily dose for 14 days was employed and no grade 4 or 5 adverse event was observed. The C_{max} of tizoxanide on days 5 and 14 were 11.3 (4.6) µg/mL and 9.0 (4.1) µg/mL, respectively[25]. Therefore, to characterise the exposure-response relationship of tizoxanide when 1000 mg nitazoxanide is taken two times daily in combination with 300/100 mg atazanavir/ritonavir, pharmacokinetic (PK) sampling will be performed in trial participants on days 5 and 14 (intensive) and on days 3, 7, 10, 21, and 28 (sparse) based on the following schedule:

Specimen	Intensive pharmacokinetic (PK) sampling (n = 12)[5]	Sparse PK sampling (n = 49)
Dried Blood Spot (DBS)	0.5, 1, 2, 4, 6, 8 and 12 hours after dose on day 5<br>0.5, 1, 2, 4, 6, 8 and 12 hours after dose on day 14	12 hours after dose on day 2<br>12 hours after dose on day 4<br>12 hours after dose on day 6<br>12 hours after dose on day 7<br>12 hours after dose on day 14<br>12 hours after dose on day 28
Saliva	0.5, 1, 2, 4, 6, 8 and 12 hours after dose on day 5<br>0.5, 1, 2, 4, 6, 8 and 12 hours after dose on day 14	12 hours after dose on day 2<br>12 hours after dose on day 7<br>12 hours after dose on day 14

14. Ethical Considerations

This trial will be conducted in compliance with the protocol, with the principles of ICH Guideline E6 for Good Clinical Practice, the Declaration of Helsinki, and all applicable regulatory requirements. Ethical clearance for the conduct of this trial has been obtained from the National Health Research Ethics Committee (NHREC), Abuja, Nigeria (approval number: NHREC/01/01/2007-26/08/2020) and the University of Liverpool's Health and Life Sciences Ethics Committee - human participants, tissues and databases (reference number: 8074). Clinical Trial Authorisation was granted by the National Agency for Food and Drug Administration and Control (NAFDAC) on 06/10/2020. Participation will be entirely voluntary and patients will be allowed to withdraw their consent at any stage. In addition, not consenting will not affect care in any way. All samples will be anonymised and retained until completely used up and no clinical data will be kept or passed on to a third party. No financial benefits will accrue to the researchers as a direct result of this study and samples may not be sold for profit, in cash or in kind.

15. Protocol Amendment

The trial protocol has been designed to meet highest level of standard that will primarily ensure safety of participants and output of good and reliable data. However, major and minor modifications can sometimes be made to remove or avoid any unanticipated risk or harm to the participant or affect the integrity of study outcomes. Whenever this happens, the amendments will be saved in a new version of the protocol, with the changes from previous version highlighted in the version history page. The amended protocol will be shared with all investigators. The revised and previously approved versions of the protocol will be submitted to the ethics committees (NHREC, Nigeria and the University of Liverpool Health and Life Sciences) and NAFDAC for approval before the changes are implemented in the trial.

[5] Both intensive and sparse PK sampling will be performed after the morning dose of 1000 mg nitazoxanide. This will also capture the C_{12} and C_{trough} of atazanavir/ritonavir which will be taken with the evening dose of 1000 mg nitazoxanide.

16. Study Monitoring

The study will be monitored by an internal Safety Review Committee (SRC), an Independent Data and Safety Monitoring Committee (IDSMC) and an Independent Medical Monitor. Because of the infection prevention and control implemented at COVID-19 treatment centres, a virtual monitoring plan will be implemented.

16.1. Safety Review Committee (SRC)

The SRC will monitor the conduct of the study through weekly summary reports of enrolment, baseline characteristics, laboratory results, adverse events, protocol deviations, reasons for premature discontinuations and adverse events. On a weekly basis, one of the investigators designated as the internal safety monitor who is also member of the SRC will review by-arm summaries of premature study discontinuations and premature study treatment discontinuations (and reasons) and AEs. A detailed description of the operational aspects of the SRC, including membership, will be documented in the SRC Charter.

16.2. Independent Medical Monitor

The independent monitor will conduct up to four visits: one pre-study, two routine and close out monitoring visits. The first routine monitoring visit will occur as soon as the first participant is recruited or within 2 weeks of the first participant is recruited. During each visit, the monitor will review the individual participant records for prospectively identified critical data and processes that if inaccurate, not performed, or performed incorrectly, would threaten the protection of human subjects or the integrity of the study results. These include verification that informed consent was obtained appropriately, adherence to protocol eligibility criteria, documentation of study drug accountability and administration, conduct and documentation of procedures and assessments related to study endpoints, protocol-required safety assessments, adverse events documentation and reporting, as well as other procedures essential to trial integrity. The independent monitor also will inspect regulatory files for all study sites to ensure compliance with regulatory requirements, and the sites' pharmacies to review product storage and management. These activities are described in the Monitoring Plan and SOP.

16.3. Independent Data and Safety Monitoring Committee (IDSMC)

The IDSMC will include individuals with expertise in respiratory viruses, antiviral therapies and shedding, emerging epidemics, and biostatistics. The IDSMC will monitor the trial for safety, operational futility, and efficacy. The IDSMC will also conduct periodic assessments of factors that can affect project implementation, including accrual, retention, performance and variation across sites. IDSMC meetings will be by teleconference. Open reports containing accrual and retention rates, participant characteristics, and serious adverse events (SAEs) will be sent to the protocol team and IDSMC members a week ahead. All safety issues will be reviewed by the IDSMC after the first 30 participants have completed 14 days of follow up and when any grade 3 or higher adverse event occurred in the intervention group. A detailed description of the operational aspects, including membership, will be documented in the IDSMB Charter.

17. Treatment Discontinuation and Withdrawal From Trial

17.1. Discontinuation

Study treatment may be discontinued for any of the following reasons:

- Request to terminate study treatment by participant

- Need to start concomitant medications known to interact with study intervention
- Development of any contraindication to study drug
- An adverse event that necessitates discontinuation of study drug
- Other reasons believed by the clinical investigators or primary care providers to place the participant at risk.

Where participants stop study intervention, they may continue in the trial off study drug with continued evaluations. The reason for study drug discontinuation will be documented.

17.2. Withdrawal from the trial

Although a participant is not obliged to explain their reason for withdrawing prematurely from a trial, reasonable effort will be made to ascertain the reason while fully respecting the participant's rights. Additionally, the investigators may withdraw a participant from the trial for any of the following reasons:

- Decision of the ethics committees or the regulatory agency as part of their duties, clinical investigators or primary care providers if they think continued participation is no longer in the best interest of the participant.
- If failure to comply with the protocol requirements by the participant puts them or the reliability of resulting data at risk.
- If a participant leaves the isolation and treatment centre (study site) against medical advice, they will be considered lost to follow-up and the following actions will be taken:
    - The site clinical investigator (or a designated member of the study team) will attempt to contact the participant as soon as possible and counsel the participant on the need to follow the national COVID-19 case management guidance.
    - The caller will ascertain whether or not the participant wishes to continue in the trial and return to the isolation and treatment centre to facilitate this. Arrangement to return participants to site will be as per existing SOP of the NCDC.
    - Where possible, up to 2 telephone call attempts per day must be made to the participants usual mobile number or their next of kin (if necessary) in the 3 days following a participant's absence from site before a participant will be deemed lost to follow-up. A participant who is unreachable after these efforts will be withdrawn from the study due to lost to follow-up.

Any data collected before such withdrawal of consent will be retained and used. Withdrawing participants will have the right to request destruction of any samples taken and not yet tested. Such requests will be documented by the site investigator. Whenever possible, follow up assessments scheduled for day 28 study exit visit will be completed at the time of withdrawal or discontinuation.

18. Data Management

18.1. Electronic data capture

Data from screening and daily follow up visits will be entered into a study specific, cloud-based electronic database using REDCap Mobile (Vanderbilt University, Nashville, Tennessee, USA) by designated staff. REDCap is a secure 21 CFR Part 11 compliant web application for building and

managing online surveys and databases. Access will be through a designated secure mobile device at each study site. Users at each site will be assigned to a Data Access Group and will only be able to see records created by users within their site. All data will be handled in accordance with the Nigeria Data Protection Regulation 2019.

18.2. Source documentation

For each participant, the following source documents will be collected and stored on site: COVID-19 test result, signed informed consent form, copies of all relevant reports and laboratory tests, comments on results and reference to any adverse events. Scanned images of these documents will be captured using study mobile device and transmitted to the database through the appropriate data collection instrument using REDCap.

18.3. Data sharing

Results from the NACOVID trial will be shared with relevant bodies and individuals in a timely manner using appropriate platforms and forums (including preprint platforms, open access research publications) to ensure usefulness for the ongoing global health emergency. Summary data will be made open access. However, request for access to disaggregated and anonymised data will be assessed on a case by case basis, depending on the qualifications of the requester, quality of request and type of secondary analysis proposed.

18.4. Archiving and retention of data

Following completion of the study, essential documents in the trial master file and data records will be retained by the sponsor for 20 years (and securely destroyed thereafter) in accordance with Good Clinical Practice and applicable regulatory requirements.

19. Availability of Data and Materials

Trial data are available to the investigators through the Obafemi Awolowo University REDCap web platform. Summary data will be made open access. However, request for access to disaggregated and anonymised data will be assessed on a case by case basis, depending on the qualifications of the requester, quality of request and type of secondary analysis proposed.

20. Role of Study Sponsor and Funder

The study sponsor and funder will not have any role in study design; data collection, management, analysis, and interpretation; report writing; and the decision to publish findings from this trial. These decisions will be made by the investigators.

21. Adverse Events Management

An adverse event (AE) is any untoward medical occurrence in a subject administered a pharmaceutical product and which does not necessarily have a causal relationship with the study treatments. An AE can therefore be any unfavourable and unintended sign (including an abnormal laboratory finding), symptom, or disease temporally associated with the use of a medicinal (investigational) product, whether or not considered related to the medicinal (investigational) product.

AEs observed by the Investigator, or reported by the subject, and any remedial action taken, will be recorded in the subject's CRF and should be verifiable in the subject's notes throughout the

study. The nature of each event, time of onset after drug administration, duration and severity will be documented together with the Investigator's opinion of the causal relationship to the treatment (unrelated, unlikely, possible, probable, and definite). The relationship to the study drugs of each AE will be assessed using the Liverpool Causality Assessment Tool in Appendix 7:

Definite: Distinct temporal relationship with drug treatment. Known reaction to agent or chemical group, or predicted by known pharmacology. Event cannot be explained by subject's clinical state or other factors.

Probable: Reasonable temporal relationship with drug treatment. Likely to be known reaction to agent or chemical group, or predicted by known pharmacology. Event cannot easily be explained by subject's clinical state or other factors.

Possible: Reasonable temporal relationship with drug treatment. Event could be explained by subject's clinical state or other factors.

Unlikely: Poor temporal relationship with drug treatment. Event easily explained by subject's clinical state or other factors.

Unrelated: The event occurs prior to dosing. Event or intercurrent illness is due wholly to factors other than drug treatment.

All subjects experiencing AEs, whether considered associated with the use of the study medication or not, must be monitored until the symptoms subside and any clinically relevant changes in laboratory values have returned to baseline, or until there is a satisfactory explanation for the changes observed. Procedures such as surgery should not be reported as AEs. However, the medical condition for which the procedure was performed should be reported if it meets the definition of an AE. For example, an acute appendicitis that begins during the AE reporting period should be reported as the adverse event and the resulting appendectomy noted on the CRF. Planned procedures such as surgery planned prior to the subject's enrolment into the study need not be reported as AEs if these are documented as planned at the screening visit. *Clinically significant* changes in physical examination and blood safety profiles should also be recorded as AEs. Severity should be recorded and graded according to the Division of AIDS Table for Grading the Severity of Adult and Paediatric Adverse Events.

All AEs, however minor[6], will be documented in the CRF whether or not the Investigator concludes the event to be related to drug treatment. The AE reporting period will be from the screening visit until the subject's final study visit. In addition, any untoward event that may occur subsequent to the reporting period that the Investigator assesses as possibly, probably or definitely related to the study drug medication should also be reported as an AE. AEs may be directly observed, reported spontaneously by the subject or by questioning the subject at each study visit. All AEs should be followed up until they are resolved or the subject's participation in the study ends (i.e. until the final CRF is completed for that subject). In addition, all serious and non-serious AEs assessed by the Investigator as possibly related to the investigational medication should continue to be followed even after the subject's participation in the study is over. Such events should be followed until resolution, or until no further change can reasonably be expected. Deaths occurring

[6] Note: There is a distinction between the gravity and the intensity of an AE. Severe is a measure of intensity; thus, a severe reaction is not necessarily a serious AE. For example, a headache may be severe in intensity but would not be classified as serious unless it met one of the criteria for serious events.

more than 30 days after the final dose, which are considered to be unrelated to the study medication, should not be reported as a Serious Adverse Event.

21.1. Serious adverse event (SAE)

A SAE is any untoward medical occurrence that at any dose:

i. Results in death
ii. Is life threatening
iii. Requires in patient hospitalisation or prolongation of existing hospitalization
iv. Results in persistent or significant disability/ incapacity, or
v. Is it a congenital anomaly/birth defect?
vi. Is medically significant, i.e. medical and scientific judgment should be exercised in deciding whether other situations should be considered serious reactions, such as important medical events that might not be immediately life threatening or result in death or hospitalization but might jeopardize the subject or might require intervention to prevent one of the other outcomes listed above.

All SAEs must be reported immediately. The SAEs should be reported immediately to the Principal Investigator (within 24 hours of a member of the study team becoming aware of the event). A SAE form should be completed, and an assessment of whether the SAE is a Suspected Unexpected Serious Adverse Reaction (SUSAR) conducted. The Principal Investigator is responsible for determining whether the SAE is a SUSAR and for reporting this in accordance with GCP and applicable regulatory requirements. All SAE must be reported in accordance with local protocols for reporting adverse study events.

22. References

1. Lu H, Stratton CW, Tang Y. Outbreak of pneumonia of unknown etiology in Wuhan, China: The mystery and the miracle. *J Med Virol*. 2020;92(4):401-402. doi:10.1002/jmv.25678

2. New-type coronavirus causes pneumonia in Wuhan: expert - Xinhua | English.news.cn. Accessed May 24, 2020. http://www.xinhuanet.com/english/2020-01/09/c_138690570.htm

3. Cucinotta D, Vanelli M. WHO Declares COVID-19 a Pandemic. *Acta Bio-Medica Atenei Parm*. 2020;91(1):157-160. doi:10.23750/abm.v91i1.9397

4. Sanders JM, Monogue ML, Jodlowski TZ, Cutrell JB. Pharmacologic Treatments for Coronavirus Disease 2019 (COVID-19): A Review. *JAMA*. Published online April 13, 2020. doi:10.1001/jama.2020.6019

5. Rosenberg ES, Dufort EM, Udo T, et al. Association of Treatment With Hydroxychloroquine or Azithromycin With In-Hospital Mortality in Patients With COVID-19 in New York State. *JAMA*. Published online May 11, 2020. doi:10.1001/jama.2020.8630

6. Yu B, Li C, Chen P, et al. Low dose of hydroxychloroquine reduces fatality of critically ill patients with COVID-19. *Sci China Life Sci*.:1. doi:10.1007/s11427-020-1732-2

7. Mehra MR, Desai SS, Ruschitzka F, Patel AN. RETRACTED: Hydroxychloroquine or chloroquine with or without a macrolide for treatment of COVID-19: a multinational registry analysis. *The Lancet*. 2020;0(0). doi:10.1016/S0140-6736(20)31180-6

8. Boulware DR, Pullen MF, Bangdiwala AS, et al. A Randomized Trial of Hydroxychloroquine as Postexposure Prophylaxis for Covid-19. *N Engl J Med*. 2020;0(0):null. doi:10.1056/NEJMoa2016638

9. No clinical benefit from use of hydroxychloroquine in hospitalised patients with COVID-19 — RECOVERY Trial. Accessed June 11, 2020. https://www.recoverytrial.net/news/statement-from-the-chief-investigators-of-the-randomised-evaluation-of-covid-19-therapy-recovery-trial-on-hydroxychloroquine-5-june-2020-no-clinical-benefit-from-use-of-hydroxychloroquine-in-hospitalised-patients-with-covid-19

10. Antinori S, Cossu MV, Ridolfo AL, et al. Compassionate remdesivir treatment of severe Covid-19 pneumonia in intensive care unit (ICU) and Non-ICU patients: Clinical outcome and differences in post_treatment hospitalisation status. *Pharmacol Res*. Published online May 11, 2020:104899. doi:10.1016/j.phrs.2020.104899

11. Hung IF-N, Lung K-C, Tso EY-K, et al. Triple combination of interferon beta-1b, lopinavir–ritonavir, and ribavirin in the treatment of patients admitted to hospital with COVID-19: an open-label, randomised, phase 2 trial. *The Lancet*. 2020;0(0). doi:10.1016/S0140-6736(20)31042-4

12. Central and South America now ‘intense zones’ for COVID-19 transmission. UN News. Published June 1, 2020. Accessed June 11, 2020. https://news.un.org/en/story/2020/06/1065252

13. Baker EH, Gnjidic D, Kirkpatrick CMJ, Pirmohamed SM, Wright DFB, Zecharia AY. A call for the appropriate application of clinical pharmacological principles in the search for safe and efficacious COVID-19 (SARS-COV-2) treatments. *Br J Clin Pharmacol*. n/a(n/a). doi:10.1111/bcp.14416

14. Rossignol J-F. Nitazoxanide, a new drug candidate for the treatment of Middle East respiratory syndrome coronavirus. *J Infect Public Health*. 2016;9(3):227-230. doi:10.1016/j.jiph.2016.04.001

15. Cao J, Forrest JC, Zhang X. A screen of the NIH Clinical Collection small molecule library identifies potential anti-coronavirus drugs. *Antiviral Res*. 2015;114:1-10. doi:10.1016/j.antiviral.2014.11.010

16. National Centre for Advancing Translational Sciences. Drug Repurposing - Tizoxanide. Accessed June 16, 2020. https://opendata.ncats.nih.gov/covid19/sample?sdid=249850958

17. Effect of Nitazoxanide in Adults and Adolescents With Acute Uncomplicated Influenza: A Double-Blind, Randomised, Placebo-Controlled, Phase 2b/3 Trial - PubMed. Accessed May 22, 2020. https://pubmed.ncbi.nlm.nih.gov/24852376/

18. Tilmanis D, van Baalen C, Oh DY, Rossignol J-F, Hurt AC. The susceptibility of circulating human influenza viruses to tizoxanide, the active metabolite of nitazoxanide. *Antiviral Res*. 2017;147:142-148. doi:10.1016/j.antiviral.2017.10.002

19. Lu R, Zhao X, Li J, et al. Genomic characterisation and epidemiology of 2019 novel coronavirus: implications for virus origins and receptor binding. *The Lancet*. 2020;395(10224):565-574. doi:10.1016/S0140-6736(20)30251-8

20. Xu J, Zhao S, Teng T, et al. Systematic Comparison of Two Animal-to-Human Transmitted Human Coronaviruses: SARS-CoV-2 and SARS-CoV. *Viruses*. 2020;12(2). doi:10.3390/v12020244

21. Arshad U, Pertinez H, Box H, et al. Prioritisation of potential anti-SARS-CoV-2 drug repurposing opportunities based on ability to achieve adequate target site concentrations derived from their established human pharmacokinetics. *medRxiv*. Published online April 22, 2020:2020.04.16.20068379. doi:10.1101/2020.04.16.20068379

22. Rajoli RK, Pertinez H, Arshad U, et al. Dose prediction for repurposing nitazoxanide in SARS-CoV-2 treatment or chemoprophylaxis. *medRxiv*. Published online May 6, 2020:2020.05.01.20087130. doi:10.1101/2020.05.01.20087130

23. FDA Centre for Drug Evaluation and Research. Medical Review - Nitazoxanide. Accessed June 16, 2020. https://www.accessdata.fda.gov/drugsatfda_docs/nda/2004/21-497_Alinia_Medr_P1.pdf

24. Romark, L.C. Nitaxoxanide (Alinia) Prescribing Information. Accessed June 16, 2020. https://www.accessdata.fda.gov/drugsatfda_docs/label/2016/021497s001,021498s004lbl.pdf

25. Walsh KF, McAulay K, Lee MH, et al. Early Bactericidal Activity Trial of Nitazoxanide for Pulmonary Tuberculosis. *Antimicrob Agents Chemother*. 2020;64(5). doi:10.1128/AAC.01956-19

26. Zulu I, Kelly P, Njobvu L, et al. Nitazoxanide for persistent diarrhoea in Zambian acquired immune deficiency syndrome patients: a randomized-controlled trial. *Aliment Pharmacol Ther*. 2005;21(6):757-763. doi:10.1111/j.1365-2036.2005.02394.x

27. Rossignol J-F. Nitazoxanide in the treatment of acquired immune deficiency syndrome-related cryptosporidiosis: results of the United States compassionate use program in 365 patients. *Aliment Pharmacol Ther*. 2006;24(5):887-894. doi:10.1111/j.1365-2036.2006.03033.x

28. Fintelman-Rodrigues N, Sacramento CQ, Lima CR, et al. Atazanavir inhibits SARS-CoV-2 replication and pro-inflammatory cytokine production. *bioRxiv*. Published online April 6, 2020:2020.04.04.020925. doi:10.1101/2020.04.04.020925

29. Fehr AR, Perlman S. Coronaviruses: An Overview of Their Replication and Pathogenesis. *Coronaviruses*. 2015;1282:1-23. doi:10.1007/978-1-4939-2438-7_1

30. Zhang D, Chando TJ, Everett DW, Patten CJ, Dehal SS, Humphreys WG. In vitro inhibition of UDP glucuronosyltransferases by atazanavir and other HIV protease inhibitors and the relationship of this property to in vivo bilirubin glucuronidation. *Drug Metab Dispos Biol Fate Chem*. 2005;33(11):1729-1739. doi:10.1124/dmd.105.005447

31. FDA. FDA Guidance on Conduct of Clinical Trials of Medical Products during COVID-19 Public Health Emergency. Published online July 2, 2020. Accessed July 7, 2020. https://www.fda.gov/media/136238/download

32. Nigeria Centre for Disease Control. National Interim Guidelines for Clinical Management of COVID-19 VERSION 2. Published online May 2020. https://covid19.ncdc.gov.ng/media/files/COVID19ClinicalCaseMgt.pdf

33. Marshall JC, Murthy S, Diaz J, et al. A minimal common outcome measure set for COVID-19 clinical research. *Lancet Infect Dis*. 2020;0(0). doi:10.1016/S1473-3099(20)30483-7

34. Powers JH, Bacci ED, Leidy NK, et al. Performance of the inFLUenza Patient-Reported Outcome (FLU-PRO) diary in patients with influenza-like illness (ILI). *PLoS ONE*. 2018;13(3). doi:10.1371/journal.pone.0194180

35. Yu J, Powers JH, Vallo D, Falloon J. Evaluation of Efficacy Endpoints for a Phase IIb Study of a Respiratory Syncytial Virus Vaccine in Older Adults Using Patient-Reported Outcomes With Laboratory Confirmation. *Value Health J Int Soc Pharmacoeconomics Outcomes Res*. 2020;23(2):227-235. doi:10.1016/j.jval.2019.09.2747

36. Wenling Wang, Yanli Xu, Ruqin Gao, et al. Detection of SARS-CoV-2 in Different Types of Clinical Specimens. *JAMA*. 2020;323(18):1843-1844.

37. Huanqin Han, Qingfeng Luo, Fan Mo, Lieming Long, Weiqiang Zheng. SARS-CoV-2 RNA more readily detected in induced sputum than in throat swabs of convalescent COVID-19 patients. *Lancet Infect Dis*. doi:https://doi.org/10.1016/S1473-3099(20)30174-2

Appendix 1. Informed Consent Process

Appendix 2. Participant Information Leaflet

Appendix 3. Informed Consent Form – Method 1

Appendix 3. Informed Consent Form – Method 2

Appendix 4. Prescription Information

a) **Nitazoxanide**[7]

b) **Atazanavir/ritonavir**[8]

Appendix 5. Clinical Management of COVID-19[9]

Appendix 6. DAIDS Adverse Events Grading Table

Appendix 7. Liverpool Causality Assessment Tool (LCAT)

Appendix 8. The inFLUenza Patient-Reported Outcome (FLU-PRO) questionnaire

[7] Full prescribing information available at: https://www.accessdata.fda.gov/drugsatfda_docs/label/2005/021818lbl.pdf

[8] Full prescribing information available at: https://www.accessdata.fda.gov/drugsatfda_docs/label/2011/021567s026lbl.pdf

[9] Source: Nigeria Centre for Disease Control. National Interim Guidelines for Clinical Management of COVID-19 Version 2. Published online May 2020. https://covid19.ncdc.gov.ng/media/files/COVID19ClinicalCaseMgt.pdf

Appendix 9. Key Trial Contact Details

Sponsor Representative	Francis Adesina, PhD<br>Professor and Executive Director, Central Office of Research, Obafemi Awolowo University, Ile-Ife, Nigeria<br>fadesina@oauife.edu.ng
Ethics Committees	Ado Danladi<br>Desk Officer, National Health Research Ethics Committee, Federal Ministry of Health, Federal Secretariat Complex, Abuja, Nigeria<br>deskofficer@nhrec.net<br><br>Research Integrity and Ethics Officer<br>University of Liverpool, Liverpool, United Kingdom<br>ethics@liv.ac.uk
Regulatory Agency	Anastasia Ofojee<br>Senior Regulatory Officer, Clinical Trials and Drug Division, Drug Evaluation and Reseach Directorate, National Agency for Food and Drug Administration and Control (NAFDAC), Lagos, Nigeria.<br>ofojee.anastasia@nafdac.gov.ng
Independent Medical Monitor	Adebola Orimadegun, MBBS, MSc Epid. (Ibadan), MSc. Clinical Trials (London), PhD (Ibadan), FWACP<br>Professor and Consultant Paediatrician, Institute of Child Health, College of Medicine, University of Ibadan, Ibadan, Nigeria<br>ae.orimadegun@ui.edu.ng
